# Supplementary material for: Quality of Life in Vulnerable Older Patients with Metastatic Colorectal Cancer Receiving Palliative Chemotherapy—The Randomized NORDIC9-Study
Source: Cancers (Basel). 2021 May 26;13(11):2604. doi: 10.3390/cancers13112604 (PMC8198682; doi:10.3390/cancers13112604)
Supplement: Supplementary file 1 [file cancers-13-02604-s001.zip › cancers-1217438-supplementary.pdf]

cancers

MDPI

Article

Quality of Life in Vulnerable Older Patients with Metastatic Colorectal Cancer Receiving Palliative Chemotherapy—The Randomized NORDIC9-Study

Gabor Liposits [1,2,3,*], Henrik Rode Eshøj [4], Sören Möller [2,5], Stine Brændegaard Winther [1,3], Halla Skuladottir [6], Jesper Ryg [2,3,7], Eva Hofsli [8,9], Carl-Henrik Shah [10], Laurids Østergaard Poulsen [11], Åke Berglund [12], Camilla Qvortrup [3], Pia Österlund [13,14,15], Bengt Glimelius [12], Halfdan Sorbye [16,17] and Per Pfeiffer [1,2,3]

Table S1. Bayesian analysis of EORTC QLQ-C30 domain measurements reporting the (posterior) probability of superiority (respectively, non-inferiority/equivalence/inferiority) of SOx compared to S1.

EORTC QLQ-C30 Domains	Change from Baseline to 9-Week Follow-Up				Change from Baseline to 18-Week Follow-Up			
	Superiority	Non-Inferiority	Equivalence	Inferiority	Superiority	Non-Inferiority	Equivalence	Inferiority
	Posterior Probability							
Global QoL	58.60%	99.95%	41.35%	0.05%	4.55%	83.06%	78.51%	16.94%
Physical functioning	49.01%	99.96%	50.95%	0.04%	54.81%	99.90%	45.09%	0.10%
Social functioning	20.84%	97.15%	76.31%	2.85%	24.84%	94.48%	69.64%	5.52%
Role functioning	16.48%	99.43%	82.95%	0.57%	18.99%	98.48%	79.49%	1.52%
Fatigue	0.28%	63.61%	63.33%	36.39%	0.37%	46.41%	46.04%	53.59%
Nausea and vomiting	1.90%	99.83%	97.93%	0.17%	0.07%	88.31%	88.24%	11.69%
Diarrhea	15.68%	89.18%	73.5%	10.82%	12.23%	76.38%	64.15%	23.62%
Pain	0.07%	95.4%	95.33%	4.6%	0.01%	76.12%	76.11%	23.88%
Dyspnea	68.64%	100%	31.36%	0.00%	39.38%	99.72%	60.34%	0.28%

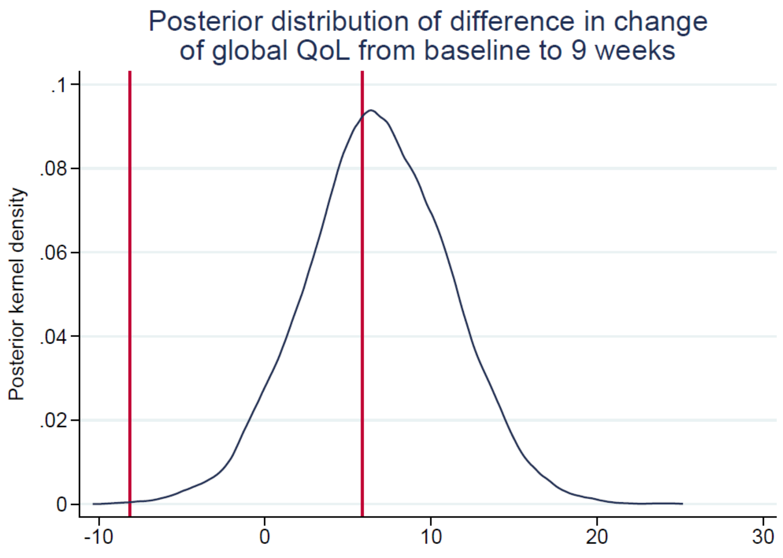

Figure S1. Posterior distribution of difference in global QoL at 9-week follow-up.
